# Response to Juberg et al.

**DOI:** 10.1186/s12940-019-0466-6

**Published:** 2019-04-03

**Authors:** Axel Mie, Christina Rudén, Philippe Grandjean

**Affiliations:** 1 Department of Clinical Science and Education, Karolinska Institutet, Södersjukhuset, 11883 Stockholm, Sweden; 2 Centre for Organic Food and Farming (EPOK), Swedish University of Agricultural Sciences (SLU), Ultuna, Sweden; 3 Department of Environmental Science and Analytical Chemistry, Stockholm University, Stockholm, Sweden; 4 Department of Public Health, University of Southern Denmark, Odense, Denmark; 5 Department of Environmental Health, Harvard T.H. Chan School of Public Health, Boston, USA

## Introduction

We appreciate the response from the pesticide producer and the test laboratory on our Commentary [1], and we are pleased to be asked for our comments. The response [2] contains some unjustified statements that we wish to call attention to.

## Cerebellar dimensions

Juberg et al. [2] interpret the presence of a statistically significant effect of chlorpyrifos at low and medium dose on cerebellar height of PND 11 pups as a processing artifact due to a prolonged time in fixative, as compared to control and high dose group brains. This new assertion of a delay in processing for certain dose groups is in contradiction to the method descriptions for the PND 11 samples [3, 4].

Furthermore, while shrinking of brain tissue during fixation in formaldehyde solutions is well known, it is not known to selectively affect the cerebellum. However, on PND 11, no other brain regional measures were affected at low and medium dose. Also, no shrinking was observed in the PND 65 female mid-dose cerebellar height, in spite of a documented longer fixation time of these samples compared to control and high dose.

Thus, the lower cerebellar height at all dose levels on PND 11 should be interpreted as treatment-related.

## Pup exposure

For chlorpyrifos, the fetal internal dose (blood concentration) is approximately a factor of 2 below the pregnant dam’s internal dose [5]. However, for PND 5 pups, the exposure of the pup via milk is a factor of 50–200 below the dam’s exposure [5, 6]. These data indicate that the nursing pups’ exposure falls below intended levels by a very substantial factor. Accordingly, during most of the brain growth spurt, pups were either exposed far below nominal exposures (PND 0–11) or not at all (from PND 12). However, neither the DNT test report [4] nor Juberg et al. [2] acknowledge this limitation. While direct dosing of pups is challenging, technical guidance [7] and specific descriptions for chlorpyrifos [6] exist.

Juberg et al. [2] indicate that early postnatal toxicokinetic data are included in a pilot study report for chlorpyrifos-methyl. These data are not included in the DNT test report, and their impact on the study conclusions are not discussed, despite test guideline requirements [8]. Knowledge regarding the adequacy of postnatal exposure is necessary for an evaluation of the study.

We note that one of the co-authors of the Juberg et al. letter [2] has contributed to the literature on the importance of the offspring’s exposure after birth [6]. We therefore find Juberg et al.’s suggestion to begin necessary direct dosing of pups only around PND 10–11 rather surprising and not in accordance with the intention of DNT tests.

## Statistical approach

When we highlighted post-hoc changes in the statistical protocol in our Commentary [1], we referred to the changes that were introduced on 9 April, 1998, i.e., after the draft report had been finalized [4]. Consequences of such post-hoc changes to the statistical protocol are not spelled out [4] but appear e.g. to have changed the conclusion regarding an effect of chlorpyrifos on motor activity at young age. Contrary to what Juberg et al. [2] suggest, the cited paper does not suggest the use of α = 0.02 [9]. We believe that it is inappropriate to use a lower-than-conventional α value in the absence of evidence that adequate statistical power is maintained. When examining in further detail the DNT study, we found a significant effect of chlorpyrifos on motor activity habituation (Dose Group x Block interaction) at high dose that was not reported [3, 4].

## Positive control

Juberg et al. [2] speculate that the absence of DNT in the positive control study using lead nitrate might be due to the fact that lead nitrate rather than lead acetate was used. However, no support for this speculation is provided. Juberg et al. [2] suggest a “lack of exposure” as a possible reason for the absence of neurodevelopmental effects. While implausible given the observed effects of lead toxicity, including deaths at high dose, this comment is counter to Juberg et al.’s own opinion on the lacking postnatal exposure to chlorpyrifos. Additional positive control data included in the DNT study report, for motor activity only, fail to meet the requirement of age-matching [10]. The test guideline [10] requires that positive control data must “demonstrate the sensitivity of the procedures being used”. There is low confidence in any null findings for chlorpyrifos because the DNT study [4] fails on these criteria for all neurodevelopmental endpoints.

## Missing data points

As Juberg et al. [2] point out, it is important that only homologous brain sections are included in morphometrics measurements. A recent test guideline highlights for brain morphometrics that “samples from fewer than 6 animals/sex/dose level would generally not be considered sufficient for the purposes of this Test Guideline” [11]. In juvenile offspring (PND 21) in the chlorpyrifos-methyl study, numbers of brain sections included for cerebellum height are insufficient by this criterion in three of four groups. However, neither the DNT study report [12] nor Juberg et al. [2] acknowledge this fact, which is only apparent when inspecting the raw data. We also note that individual images for assessment of the homology of sections used for morphometry are, contrary to guideline requirements [8], not included in the test report [12].

## Adverse effects in humans

Juberg et al. [2] claim that “many experts do not agree” with a recent assessment of developmental neurotoxicity in humans and refer to a commentary supported by the American Chemistry Council [13]. A more appropriate reference is the recent review carried out by key scientists who have conducted research on developmental neurotoxicity in children [14]. This review emphasized the clear preponderance of documentation showing a neurotoxic risk from exposures to organophosphate pesticides, such as chlorpyrifos. The assessment of costs within the EU carried out by international colleagues [15] emphasized that the final cost estimates are influenced by parameters such as attributable fractions and exposure levels and that both under- and overestimations are possible. In agreement with this assessment, Dutch researchers recently concluded: “The highest cost of EDC-associated health effects, are found in the group of neurobehavioral diseases, disorders and cognitive conditions. This group of neurobehavioral disorders includes several pervasive disorders that remain during a person’s whole lifetime, thereby resulting in substantial costs” [16].

We believe that prevention of such adverse effects is of crucial importance to public health. Reliable and valid safety tests are one necessary prerequisite.

A recent decision from the Court of Justice of the European Union [17] will improve access to all pesticide toxicity studies that EU authorities rely on. That would be a welcome development enabling academic scientists and others to scrutinize these safety tests.

## Abbreviations

DNT: Developmental neurotoxicity
EDC: Endocrine disrupting compound
EU: European Union
PND: Postnatal day
