# Correction to: Response to Juberg et al

**DOI:** 10.1186/s12940-020-00633-7

**Published:** 2020-07-08

**Authors:** Axel Mie, Christina Rudén, Philippe Grandjean

**Affiliations:** 1 Department of Clinical Science and Education, Karolinska Institutet, Södersjukhuset, 11883 Stockholm, Sweden; 2 Centre for Organic Food and Farming (EPOK), Swedish University of Agricultural Sciences (SLU), Ultuna, Sweden; 3 Department of Environmental Science and Analytical Chemistry, Stockholm University, Stockholm, Sweden; 4 Department of Public Health, University of Southern Denmark, Odense, Denmark; 5 Department of Environmental Health, Harvard T.H. Chan School of Public Health, Boston, USA

**Correction to: Environ Health 18, 29 (2019)**

**https://doi.org/10.1186/s12940-019-0466-6**

In the Letter to Editor Response [1], we made a statement that the article by Bond and Dietrich [2], referred to by Juberg et al. [3], was supported by American Chemistry Council (ACC). It has been brought to our attention that the current wording suggests that the particular work by Bond and Dietrich [2] received financial or other support from ACC, which was not the intention of the authors. The statement was intended to acknowledge that the ACC has expressed its support of their paper [4]. We wish to amend our published response as follows in order to clarify this:

Juberg et al. [3] claim that “many experts do not agree” with a recent assessment of developmental neurotoxicity in humans and refer to an article by Bond and Dietrich (2017) [2].

## References

[1] Mie (2019). Response to Juberg et al. Environ Health.

[2] Bond G, Dietrich DR (2017). Human cost burden of exposure to endocrine disrupting chemicals. A critical review. Arch Toxicol.

[3] Juberg D, Hoberman AM, Marty S, Picut CA, Stump DG (2018). Letter to the editor re: commentary in environmental health titled “safety of safety evaluation of pesticides: developmental neurotoxicity of chlorpyrifos and chlorpyrifos-methyl” by Mie et al.

[4] The University of Konstanz (2017). Trasande-led human health impact and cost estimates attributed to endocrine disrupting chemical exposure completely unfounded, researchers show.

